# Surgeon assessment of significant rectal polyps using white light endoscopy alone and in comparison to fluorescence-augmented AI lesion classification

**DOI:** 10.1007/s00423-024-03364-2

**Published:** 2024-06-01

**Authors:** Niall P. Hardy, Alice Moynihan, Jeffrey Dalli, Jonathan P. Epperlein, Philip D. McEntee, Patrick A. Boland, Peter M. Neary, Ronan A. Cahill

**Affiliations:** 1 UCD Centre for Precision Surgery, University College Dublin, Dublin, Ireland; 2 IBM Research Europe, Dublin, Ireland; 3 Department of Surgery, University Hospital Waterford, University College Cork, Cork, Ireland; 4 Department of Surgery, Mater Misericordiae University Hospital, 47 Eccles Street, Dublin 7, Ireland

**Keywords:** Rectal polyps, TAMIS, Fluorescence, Artificial Intelligence, Inter-rater variability

## Abstract

**Purpose:**

Perioperative decision making for large (> 2 cm) rectal polyps with ambiguous features is complex. The most common intraprocedural assessment is clinician judgement alone while radiological and endoscopic biopsy can provide periprocedural detail. Fluorescence-augmented machine learning (FA-ML) methods may optimise local treatment strategy.

**Methods:**

Surgeons of varying grades, all performing colonoscopies independently, were asked to visually judge endoscopic videos of large benign and early-stage malignant (potentially suitable for local excision) rectal lesions on an interactive video platform (Mindstamp) with results compared with and between final pathology, radiology and a novel FA-ML classifier. Statistical analyses of data used Fleiss Multi-rater Kappa scoring, Spearman Coefficient and Frequency tables.

**Results:**

Thirty-two surgeons judged 14 ambiguous polyp videos (7 benign, 7 malignant). In all cancers, initial endoscopic biopsy had yielded false-negative results. Five of each lesion type had had a pre-excision MRI with a 60% false-positive malignancy prediction in benign lesions and a 60% over-staging and 40% equivocal rate in cancers. Average clinical visual cancer judgement accuracy was 49% (with only ‘fair’ inter-rater agreement), many reporting uncertainty and higher reported decision confidence did not correspond to higher accuracy. This compared to 86% ML accuracy. Size was misjudged visually by a mean of 20% with polyp size underestimated in 4/6 and overestimated in 2/6. Subjective narratives regarding decision-making requested for 7/14 lesions revealed wide rationale variation between participants.

**Conclusion:**

Current available clinical means of ambiguous rectal lesion assessment is suboptimal with wide inter-observer variation. Fluorescence based AI augmentation may advance this field via objective, explainable ML methods.

## Introduction

The management of complex rectal lesions is challenging and begins at index endoscopic identification. Whilst more advanced lesions (e.g. T2-T4 lesions) mandate formal bowel resection either robotically, laparoscopically or via a traditional open approach, multiple less invasive modalities now exist to manage larger benign lesions and even early-stage cancers [1]. Examples of such techniques include endoscopic mucosal resection (EMR) and endoscopic submucosal dissection (ESD) and transanal approaches such as transanal resection of tumour (TART), transanal endoscopic microsurgery (TEMS) and transanal Minimally Invasive Surgery (TAMIS)(which can offer either submucosal or full thickness excisions) [2]. 

The usefulness of such techniques is significantly limited however by the current state of the art regarding patient selection. Tissue biopsy may under sample lesions and so is inaccurate in up to 30% of those > 2 cm in size, and its results are only available sometime after the procedure [3]. Radiological imaging is also only available outside of endoscopy, with magnetic resonance imaging (MRI) often over staging lesions and endoanal ultrasound only being available where expertise permits [4]. Surface spectral illumination such as narrow band imaging (NBI) needs expertise to be helpful. As a result, the clinical management of big polyps remains strongly dependent on individual physician judgement with a resulting wide variation in the modalities of treatments offered to patients.

Whilst several commercially available artificial intelligence (AI) based adjuncts now exist to aid in real-time polyp detection at endoscopy, these technologies focus entirely on locating lesions of all sizes leaving polyp characterisation entirely up to the operator [5]. A new AI, indocyanine green (ICG) fluorescence angiography-based technology has been developed to perform real time characterisation of rectal lesions exploiting flow differentials between healthy, benign and malignant tissues with sensitivity and specificities in excess of 90% and 80% respectively [6, 7]. In this study, we sought to assess surgeon endoscopist ability to characterise significant rectal lesions through an online survey comprising white light videos and still images of ambiguous rectal lesions encountered at the time of diagnostic/therapeutic endoscopic procedures, and compare their visual judgement assessment alongside endoscopic biopsy and MRI to fluorescence-augmented AI based classification with final pathology as ground truth.

## Methods

### Study videos and participant selection

Fourteen white light endoscopic videos of significant rectal tumours with ambiguous features gathered as part of a larger prospective, multicentre, multinational trial (NCT 04220242) were chosen for this study following full Institutional Review Board (IRB) ethical approval (Approval number: 1/378/2092). Rectal lesions of consenting patients were either early-stage cancers (≤ T2) found at colonoscopy, or large (> 2 cm) benign lesions unsuitable for flexible endoscopic resection due to either their position or macroscopic features suspicious of invasive malignancy. Lesions included were those potentially amenable for full thickness local resection (e.g. by TAMIS) and were specifically chosen from the larger available bank of polyps due to their mixed benign/malignant appearances. No instruction regarding endoscopic biopsy techniques were given to proceduralists to ensure that results obtained represented “real-world” biopsy yields. Videos were obtained whilst lesions were undergoing local excision suitability assessment using a commercially available transanal access platform and Pinpoint Endoscopic near infrared Fluorescence System (Stryker Corp, Kalamazoo, MI, USA) (see Table 1 for rectal lesion demographics), with recordings made of both the white light appearances and a minimum of 120 s duration of near infrared (NIR) ICG inflow and early outflow following intravenously administered ICG (0.25 mg/kg) for comparative machine learning (ML) algorithmic analysis, as previously reported [8, 9].

**Table 1:** Patient demographics and lesion data

**Rectal Lesion Patient Characteristics**	***N*** **= 14 cases**
Male: Female	12 (86%):2 (14%)
Age in years (Mean ± Std dev)	64 ± 10.9
Benign: Malignant	7 (50%):7 (50%)
Mean lesion size (range)	44 mm (19–120 mm)
**Rectal Lesion Staging**	
Low Grade Dysplasia	5 (36%)
High Grade Dysplasia	2 (14%)
T1	5 (36%)
T2	2 (14%)

White light videos of up to 30 s duration alone (including long distance overview as well as close up views) were shown to participants for their consideration using an interactive collaborative video platform (www.mindstamp.io). Participants were recruited by inviting surgical endoscopy colleagues via our department’s clinical network. The required participant sample size was calculated by power testing as per *Jones et al.*, assuming an AI classifier reference sensitivity (established in prior works) of 93%, specificity of 85 % and statistical significance calculated at a 95% confidence interval [10]. For this study, such power testing identified 14 and 28 participants, each completing 14 polyp assessments, as the number needed to compare to the previously established ML classifier sensitivity and specificity performance.

### Study format and analysis of participant clinical assessment

Identified, consenting individuals were provided with an internet link to complete the study at their convenience using the online platform Mindstamp (Melbourne, Florida, United States). This software enables viewing and annotation of procedural video along with the record of additional participant data, including responses to defined questions. The video presentation commenced with a brief introductory segment followed by a questionnaire (level of training/practice, how often the individual regularly performed colonoscopies and if they had a special interest in complex polyp management). Participants then viewed fourteen intraprocedural videos of the selected rectal lesions and were asked to judge each lesion as benign or malignant as well as to indicate their certainty of decision (ranging from ‘no confidence’ to ‘highly confident’) using a validated confidence score for decision making in clinical situations [11]. Mean accuracy, sensitivity and specificity of judgements were calculated based on respondent answer and reported as a whole, as well as stratified by level of training/position held. Inter-rater agreement for polyp nature was calculated using Fleiss Kappa statistics and self-reported decision confidence levels were recorded on a polyp-by-polyp basis using frequency tables generated using SPSS Statistics v 27 (IBM, NY, US). Kappa agreement scores < 0.20 were considered ‘*poor*’, 0.21–0.40 ‘*fair*’, 0.41–0.60 ‘*moderate*’, 0.61–0.80 ‘*good*’ and 0.81–1.0 ‘*very good*’ [12]. Spearman’s rank-order correlation determined the relationship between confidence of a participant’s answer and the probability of it being correct, as well as the correlation between answer confidence and level of training. In six lesions, participants were asked to estimate polyp size with results reported using mean and range values (mm) and compared to measurements from the final resected pathology. Multiple viewings as well as change of mind were permitted with the candidate’s final answer being used. Participants were also asked to justify their decision (benign or malignant) using a short free-text explanation in 7/14 cases with a hierarchical based visual summary of these subjective identifying terms used to create a word cloud using dedicated software (www.wordclouds.com, Zygomatic, Vianen, The Netherlands), grouping most prevalent words overall and for benign and malignant lesions by size with increasing font size indicating increased frequency of use. A literature search was performed for commonly reported endoscopic visual features of malignancy within colorectal polyps and compared to the descriptions used by the participants.

### Computer vision and region of interest (ROI) ML classification

**Fig. 1:**
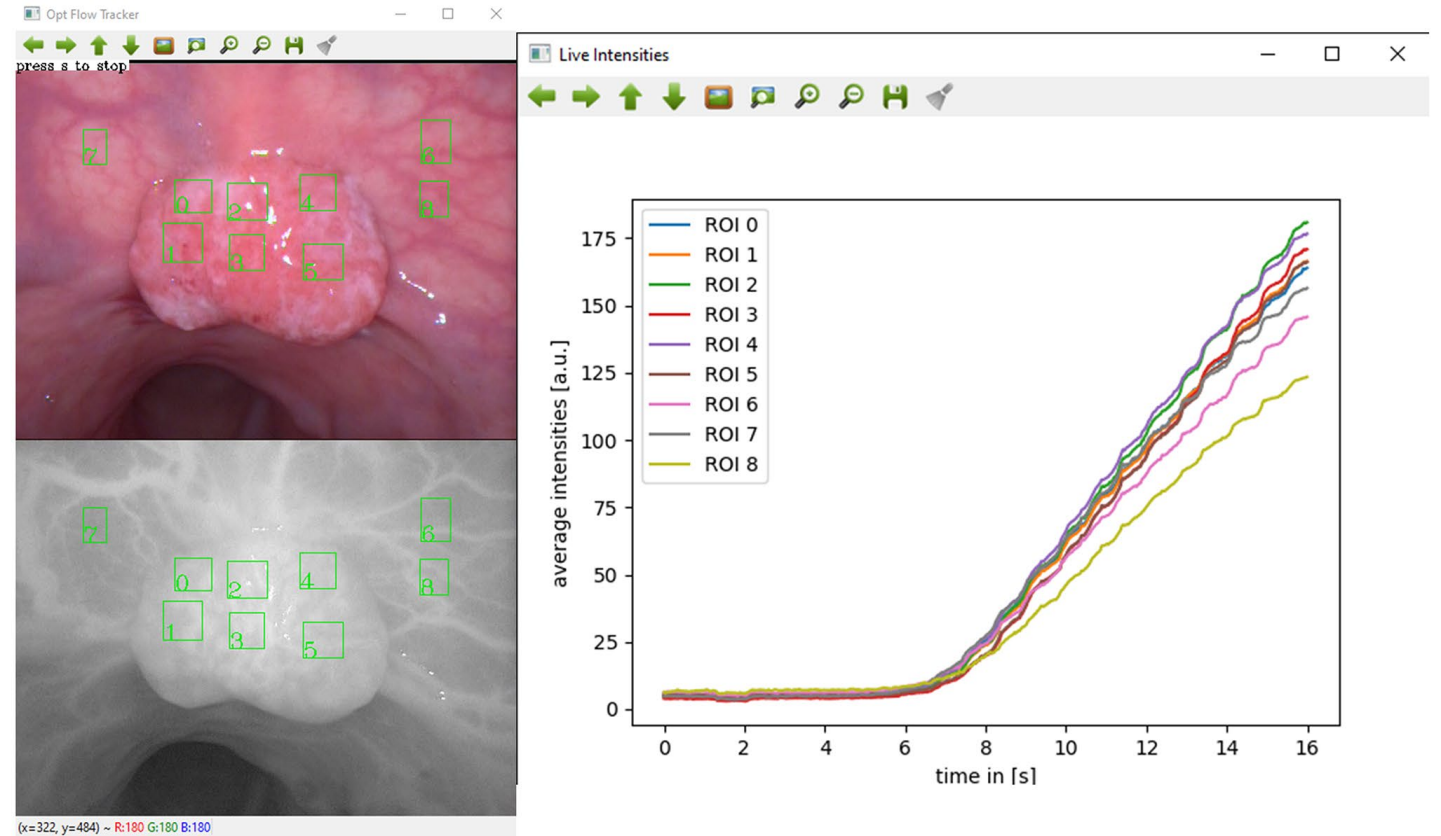
Time-fluorescence curve extraction from user-selected Regions of Interest (ROI) for algorithmic analysis. White light image (top left) used for ROI identification and computer vision tracking with extraction of fluorescence intensities from equivalent near infrared regions (bottom left). Values extracted at 30 frames per second and displayed as fluorescence-time series (right)

For the ML classification, all fourteen videos were annotated (mapping both normal and abnormal tissue within the imagery) and time-NIR fluorescence intensity curves extracted for multiple user-selected regions of interest (ROI) via a bespoke open-source tracker (IBM Research, available at https://github.com/IBM/optflow-region-tracker) utilizing the white light video source for tracking and at 30 frames per second as previously described(see Fig. 1) [9, 13, 14]. Known discriminant curve milestones including time to peak intensity, upslope, downslope, skew and centre of mass (weighted average of fluorescence intensity over time) were extracted from the curves and classified. The classifier was developed and trained on a balanced (with respect to cancer and benign ROIs) dataset comprising 32 polyps collected as part of a larger prospective study (NCT 04220242) using MATLAB ML software.

## Results

### Analysis of surgeon judgement

Thirty-two surgeons of varying grades, but all performing colonoscopies regularly (> 100 per year), completed the questionnaire for all 14 polyps resulting in 448 separate lesions assessments (see Table 2 for respondent demographics). A special interest in the management of complex rectal lesions was declared by 19/32 (60%). Regarding the lesions chosen from the archive for the study, all malignant lesions had at least one set of falsely negative endoscopic biopsies prior to definitive malignant diagnosis (either with repeat biopsy or after formal resection). Five of seven benign lesions underwent preoperative MRI with over staging as malignancy occurring in three (60%). Five of seven ultimately malignant lesions underwent pre-operative MRI imaging with over staging by at least one T-stage occurring in three and the final radiological report equivocal as to the presence of malignancy in the remaining two.

**Table 2:** Results (accuracy, sensitivity and specificity) of participant polyp (*n* = 14) assessment including stratification by level of training with comparison to AI fluorescence classifier prediction and conventional endoscopic biopsy

Group	Mean Accuracy (Range)	Mean Sensitivity (Range)	Mean Specificity Range
All (*n* = 32)	49% (21–71)	47% (17–83)	48% (0–75%)
Consultant (*n* = 13)	47% (17–83)	53% (25–75)	51% (29–71)
Late Training (> 5 years specialty practice) (*n* = 9)	56% (17–83)	49% (13–75)	52% (36–64)
Middle Training (3–5 years clinical practice) (*n* = 10)	47% (33–67)	39% (0–63)	42% (21–57)
Interest in complex polyp management (*n* = 19)	46% (17–83)	49% (13–75%)	47% (21–64)
AI Fluorescence Classifier (ROI)	87%	100%	77%
Endoscopic Biopsy	50%	0%	100%

Mean accuracy, sensitivity and specificities for the visual judgements of all 14 polyps are displayed in Table 2. Fleiss’ multi-rater kappa showed a ‘*fair*’ agreement between all 32 surveyed raters, κ = 0.261 (95% CI, 0.237 to 0.285), *p* < 0.001 with only modest variations seen between consultant (κ = 0.298 (95% CI, 0.239 to 0.358), *p* < 0.001), middle-stage training (κ = 0.317 (95% CI, 0.239 to 0.0.395), *p* < 0.001) and complex polyp interest (κ = 0.256 (95% CI, 0.216 to 0.296), *p* < 0.001) groupings and falling into ‘*poor*’ (< 0.20) for those in the late-stage training category (κ = 0.187 (95% CI, 0.100 to 0.275), *p* < 0.001).

Certainty of participant decision making, demonstrated via Likert scale frequency tables, are shown on a polyp-by-polyp basis in Table 3 with Spearman rank-order correlation showing no significant correlation between answer confidence and correctness (r_s_= -0.01, *p* = 0.833) but did demonstrate a statistically significant positive “*weak”* correlation between answering confidence and level of training (r_s_= 0.152, p = 0.001).

**Table 3:** Polyp-by-polyp breakdown of Likert decision confidence scores and majority decision vs. final pathology

Polyp no. (Final Pathology)	Majority answer	Mean Likert score	Likert Scale by Case				
			No Confidence (1)	Little Confidence (2)	Some Confidence (3)	Confident (4)	Highly Confident (5)
1 (Benign)	Benign (53%)	3.28	0 (0%)	4 (12.5%)	16 (50%)	11 (34.4%)	1 (3.1%)
2 (Cancer)	Cancer (69%)	3.38	0 (0%)	2 (6.3%)	17 (53.1%)	12 (37.5%)	1 (3.1%)
3 (Cancer)	Benign (72%)	3.06	1 (3.1%)	5 (15.6%)	17 (53.1%)	9 (28.1%)	0 (0%)
4 (Benign)	Cancer (75%)	3.59	0 (0%)	1 (3.1%)	14 (43.8%)	14 (43.8%)	3 (9.4%)
5 (Benign)	Benign (78%)	3.16	1 (3.1%)	5 (15.6%)	16 (50%)	8 (25%)	2 (6.3%)
6 (Cancer)	Benign (63%)	3.22	0 (0%)	6 (18.8%)	15 (46.9%)	9 (28.1%)	2 (6.3%)
7 (Benign)	Cancer (91%)	3.53	0 (0%)	4 (12.5%)	12 (37.5%)	11 (34.4%)	5 (15.6%)
8 (Cancer)	Cancer (91%)	3.84	0 (0%)	1 (3.1%)	9 (28.1%)	16 (50%)	6 (18.8%)
9 (Benign)	Benign (75%)	3.13	1 (3.1%)	8 (25%)	10 (31.3%)	12 (37.5%)	1 (3.1%)
10 (Cancer)	Benign (63%)	3.31	1 (3.1%)	4 (12.5%)	14 (43.8%)	10 (31.3%)	3 (9.4%)
11 (Benign)	Benign (73%)	2.94	0 (0%)	12 (37.5%)	12 (37.5%)	6 (18.8%)	2 (6.3%)
12 (Benign)	Cancer (97%)	3.53	0 (0%)	3 (9.4%)	13 (40.6%)	12 (37.5%)	4 (12.5%)
13 (Cancer)	Benign (78%)	2.91	1 (3.1%)	9 (28.1%)	15 (46.9%)	6 (18.8%)	1 (3.1%)
14 (Cancer)	Even (50%)	3.16	0 (0%)	7 (21.9%)	16 (50%)	6 (18.8%)	3 (9.4%)

Estimations of polyp sizes (*n* = 6) compared to final pathology measurements are shown in Table 4 with an average error of 20% in size estimation (versus actual) for all six polyp videos requesting measurement. The largest estimation error was in an extensive lesion not wholly visible with any single camera view with the average surgeons’ size estimate here 50% smaller than the actual lesion size. All other measured lesions were fully visualised in a single frame with an average error of only 15% in this group of five (three were judged, on average, smaller than actual measurement, and two larger). 2/6 lesions were correctly estimated, on average, to within 2 mm of actual size.

**Table 4:** Results of lesions size estimation by polyp and with breakdown of mean and range estimated by level of training. (*actual size taken from final excisional histopathological report)

Lesion Size Assessment			
Polyp Number	Group	Mean estimate (range) in mm	Actual Size*
1 (Benign)	All	60 (30–150)	120 mm
	Consultant	54 (30–100)	
	Late Training	57 (30–100)	
	Middle Training	72 (30–150)	
	Polyp Interest	59 (30–100)	
2 (Cancer)	All	20 (10–40)	29 mm
	Consultant	21 (12–40)	
	Late Training	19 (10–30)	
	Middle Training	21 (10–30)	
	Polyp Interest	23 (12–40)	
3 (Benign)	All	44 (14–70)	46 mm
	Consultant	45 (30–60)	
	Late Training	38 (20–70)	
	Middle Training	46 (14–70)	
	Polyp Interest	45 (30–70)	
4 (Benign)	All	37 (15–80)	30 mm
	Consultant	39 (25–80)	
	Late Training	35 (20–50)	
	Middle Training	35 (15–50)	
	Polyp Interest	38 (20–80)	
5 (Benign)	All	20 (10–50)	18 mm
	Consultant	17 (10–30)	
	Late Training	21 (10–50)	
	Middle Training	21 (12–50)	
	Polyp Interest	20 (10–50)	
6 (Cancer)	All	26 (8–80)	24 mm
	Consultant	29 (12–80)	
	Late Training	25 (15–60)	
	Middle Training	24 (8–40)	
	Polyp Interest	30 (14–80)	

**Fig. 2:**
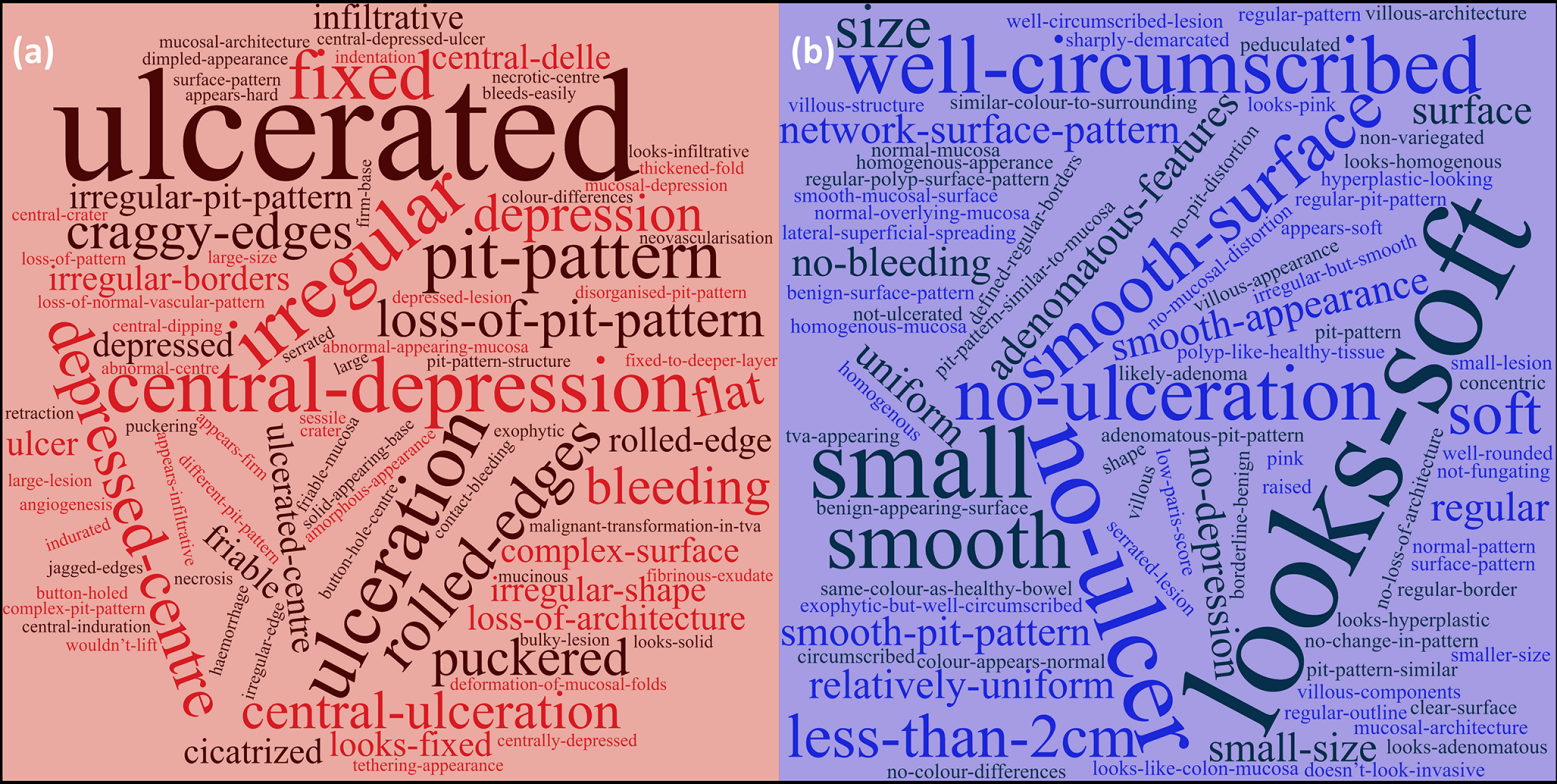
Word plot (www.wordclouds.com, Zygmomatic, Vianen, the Netherlands) of participant responses narrating their subjective approach to lesion determination. Font size corresponds to frequency of word/term use with increasing font size representing increased use. Colour and direction/orientation of words are purely for ease of artistic representation. (**a**) represents justification terminology used for participants when describing rational for lesions presumed malignant and (**b**) lesions presumed benign

Seven commonly reported white light endoscopic features of malignancy were identified from the published literature including; tumour excavation/depression, stalk swelling, large tumour size, converging tumour folds, bleeding ulcers, invasive pit pattern and non-lifting sign [15]. All terms were referenced extensively in the participant descriptions of decision making however the group majority decision regarding lesion nature was correct in only 2/7 lesions where a narrative on decision making was sought indicating that the application of such features did not help with lesion characterisation. Word clouds depicting the participant reported subjective approaches to determining lesion status are shown in Fig. 2a (cancer) and 2b (benign).

### Computer vision and region of interest (ROI) ML classification

**Fig. 3:**
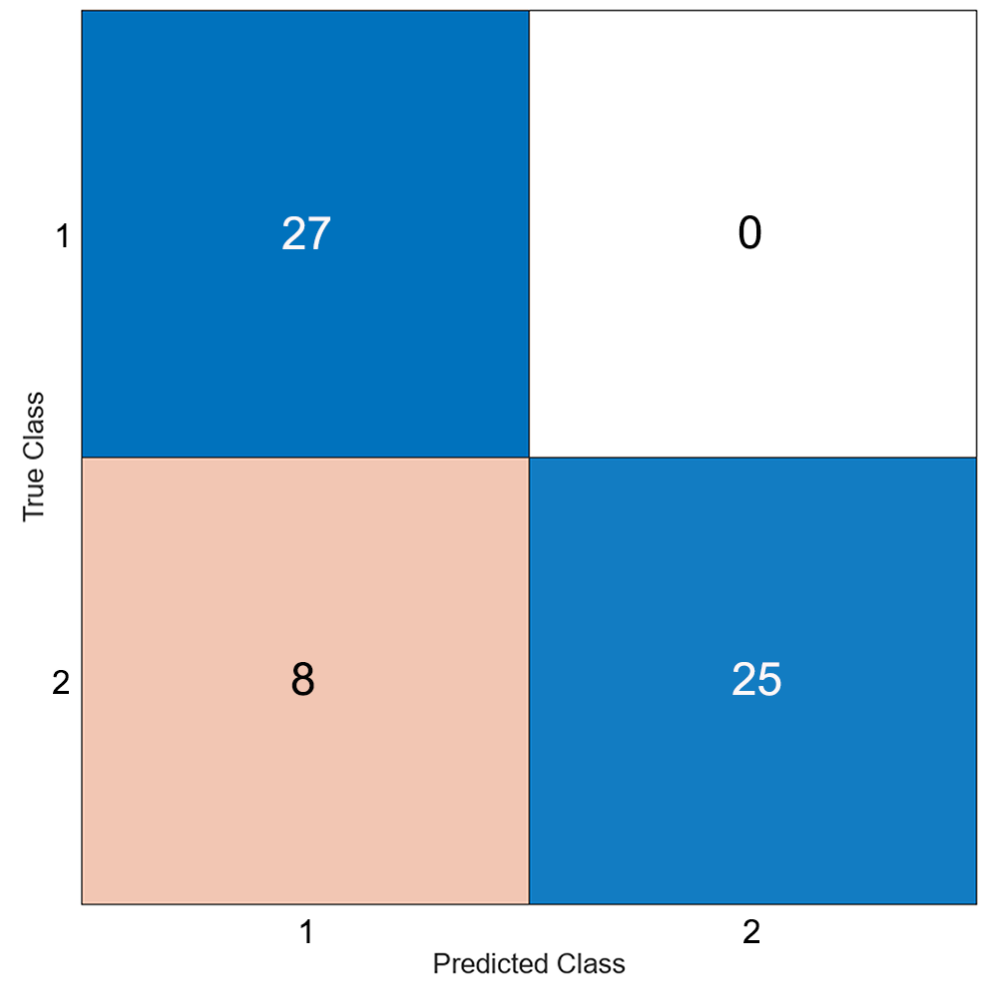
Result of machine learning classifier on 14 assessed lesions and results presented as a confusion matrix

An optimizable ensemble classifier yielded a 91.7% average accuracy for ROI classification in the 32-polyp training set using 5-fold cross validation. Subsequent ROI-based time-fluorescence curve algorithmic analysis on the 14 unseen study lesions proved 87% accurate at ROI level (60 ROIs were analysed) and 86% accurate at patient level with 2 false positive cases (both high grade dysplastic lesions, one close to the anal verge with adjacent tissue compressed by the access platform and one a regrowth having been previously locally excised) (see Fig. 3).

## Discussion

The management of rectal lesions has traditionally been divided into those lesions suitable for endoscopic excision and those requiring bowel resection in the form of a total mesorectal excision, with or without defunctioning loop ileostomy [16]. More recently, intermediate (extensive dysplastic and early-stage malignancies) lesions have been increasingly managed through local resection strategies such as ESD, EMR, TEMS and TAMIS [2]. Whilst this has allowed more patients to undergo organ preserving strategies, the importance of pre-operative patient selection has been emphasised. Piecemeal excision, although not optimal, is an acceptable outcome in dysplasia but compromises oncological principles in the presence of malignancy. Furthermore, local excision in malignancy can be insufficient in T2 or above as it does not permit lymph node status assessment, the predominant prognostic factor in non-metastatic disease, with the risk of lymph node metastasis increasing with submucosal invasion depth [17, 18]. Tumour size also represents an important factor for malignancy as well as local recurrence risk and lymph node positivity with tumours up to 3 cm generally considered lower risk [19–21]. 

The lesions in this study were chosen as they highlight the challenges associated with case selection, comprising a combination of smaller malignant lesions as well as more extensive benign lesions. The real-world clinical challenge is further highlighted in these cases given that 7/7 malignant lesions were not found to be malignant on initial sampling, with MRI techniques failing to add value also. Whilst techniques such as “bite-on-bite” sampling has been suggested to increase diagnostic yield, operators were not instructed regarding sampling methods and routine practices were followed [22]. Furthermore, such aggressive biopsy methods have been proposed to cause increased levels of post-biopsy fibrosis that may complicate subsequent local excision [23]. Endoscopic ultrasound (EUS) and even more advanced techniques such as ultrasound localization microscopy may add benefit but are not widely available, costly and resource intensive [24, 25]. 

Mean accuracy, sensitivity and specificity scores for clinical assessment approximated 40–50% across all subgroups (Table 2) suggesting that white light interpretation of such lesions is not reliable irrespective of experience or training level. ‘*Poor*’ and ‘*fair*’ kappa agreement scores across all groups also suggest that all participants experienced difficulty discerning the neoplastic status consistently across all polyps. Indeed, the consensus opinion was only correct in 6/14 lesions and opinion divided equally in 1/14. In 2/3 polyps where the majority answer exceeded 90% agreement, the consensus was incorrect (polyps 4 and 12, Table 4) and 3 of the top 4 polyps, with respect to mean Likert scores, were judged incorrectly by majority, suggesting no correlation between the confidence of participant decision and probability of a correct prediction. Not unexpectedly, confidence was found (albeit weakly) to correlate with increasing experience however the persistence of a poor accuracy should reinforce the challenges and pitfalls of dealing with these borderline lesions even for the more experienced clinicians.

As mentioned, lesion size assessment is a valuable predictor of nature with mis-sizing of lesions common where a tendency to overestimate lesion size in smaller lesions has been reported previously in studies assessing surveillance scheduling after colonoscopy [26, 27]. In this study 5/6 polyps where size judgements were provided had a mean guess within 10 mm of the final pathology size and 2/6 within 2 mm suggesting that size characteristics can be accurately judged in wholly visible lesions. Of note however the sizes quoted as “actual” did not allow for formalin shrinkage which may be up to 25% [28]. 

The endoscopic white light features of malignancy such as excavation/depression, ulceration, contact bleeding, fold convergence and invasive pit patterns have been well described and were referenced extensively by the study participants (Fig. 2) [15, 29, 30]. As evidenced by the results however, despite being associated in the literature with malignancy, these terms were not reliably matched to final pathology suggesting either misapplication of terminology, insufficiency of such features to accurately depict early stage malignancy, or both [15]. 

*Aadam et al.*, have demonstrated similar findings (regarding clinician accuracy) with a smaller number of predominantly benign polyps (five benign and one malignant) and also demonstrated limited benefit to the addition of NBI, often proposed as a solution to polyp characterisation, in non-expert hands [31]. Other techniques such as confocal laser endomicroscopy, chromoendoscopy and pit pattern analysis have shown benefit in specialist centres but are either yet to be widely adopted, with most lower gastrointestinal endoscopy being performed in non-specialist centres, or of low added value in inexperienced hands [32–34]. 

One potential solution proposed here utilises already widely established fluorescence imaging in combination with ML methods to automate the comparative analysis of ICG flow through tissue. Surgeon-selected ROIs permits extraction of time-fluorescence perfusion profiles of both healthy and tumour tissue at the time of intravenous ICG administration with significantly altered patterns noted in malignancy. This approach has been shown to yield high levels of accuracy with a large scale multi-national study (CLASSICA) ongoing to prove generalizability as well as investigate potential for intra-operative margination currently recruiting [7, 35]. Utilizing this methodology 5/7 benign lesions were successfully identified with two false positive results in a low-lying lesion at the anal verge with resulting tissue compression by the TAMIS access port as well as a previously excised lesion regrowth likely distorting blood flow in the region.

Limitations of this study include the format used to assess participants with an online video platform not permitting users to assess the lesions in an individualised manner. There is also a loss of tactile scope/instrument feedback as well as other adjuncts such as narrow band imaging (in appropriately trained individuals) and digital rectal examination which can be useful in lower rectal lesions. A number of individuals also mentioned in the narrative description of lesion assessment that they would utilise submucosal saline injection to attempt to “lift” lesions as part of their assessment [36]. 

We acknowledge that significant selection bias exists within this study by design with inclusion criteria selecting out the most challenging lesions and therefore resulting in the relatively low accuracy seen that is not representative of transanal surgery in its entirety. Lesions of these nature however comprise a large percentage of lesions encountered by those specialising in transanal surgery with the 14 included lesions comprising a significant proportion of the 100 tumours recruited as part of already mentioned study (NCT 04220242). We therefore feel that such lesions, given their frequency, warrant individual address. Furthermore, such lesions represent the cohort in which the most significant improvements can be made utilizing AI methods, as highlighted in this manuscript. Finally, although ICG recordings of the lesions were taken to permit ML assessment, we did not show these recordings to the participants as visual assessment of dynamic fluorescence perfusions patterns within neoplasia is not a currently utilised method and even in widely used applications such as intestinal perfusion, significant inter user variability exists [37, 38]. 

In conclusion, extensive benign lesions and early-stage malignancies represent a significant diagnostic challenge even in those clinically experienced with current diagnostic approaches requiring further improvement. Fluorescence based ML methods may provide further useful information without requiring specialist equipment or expertise.

## Data Availability

Anonymised data available upon reasonable request.
